# Perfluoroalkyl Acid Concentrations in Blood Samples Subjected to Transportation and Processing Delay

**DOI:** 10.1371/journal.pone.0137768

**Published:** 2015-09-10

**Authors:** Cathrine Carlsen Bach, Tine Brink Henriksen, Rossana Bossi, Bodil Hammer Bech, Jens Fuglsang, Jørn Olsen, Ellen Aagaard Nohr

**Affiliations:** 1 Perinatal Epidemiology Research Unit, Aarhus University Hospital, Aarhus, Denmark; 2 Department of Pediatrics, Aarhus University Hospital, Aarhus, Denmark; 3 Department of Environmental Science, Aarhus University, Roskilde, Denmark; 4 Section for Epidemiology, Department of Public Health, Aarhus University, Aarhus, Denmark; 5 Department of Obstetrics and Gynecology, Aarhus University Hospital, Aarhus, Denmark; 6 Department of Epidemiology, Fielding School of Public Health, University of California Los Angeles, California, United States of America; 7 Research Unit for Obstetrics and Gynecology, Institute of Clinical Research, University of Southern Denmark, Odense, Denmark; University of Cincinnati, UNITED STATES

## Abstract

**Background:**

In studies of perfluoroalkyl acids, the validity and comparability of measured concentrations may be affected by differences in the handling of biospecimens. We aimed to investigate whether measured plasma levels of perfluoroalkyl acids differed between blood samples subjected to delay and transportation prior to processing and samples with immediate processing and freezing.

**Methods:**

Pregnant women recruited at Aarhus University Hospital, Denmark, (n = 88) provided paired blood samples. For each pair of samples, one was immediately processed and plasma was frozen, and the other was delayed and transported as whole blood before processing and freezing of plasma (similar to the Danish National Birth Cohort). We measured 12 perfluoroalkyl acids and present results for compounds with more than 50% of samples above the lower limit of quantification.

**Results:**

For samples taken in the winter, relative differences between the paired samples ranged between -77 and +38% for individual perfluoroalkyl acids. In most cases concentrations were lower in the delayed and transported samples, e.g. the relative difference was -29% (95% confidence interval -30; -27) for perfluorooctane sulfonate. For perfluorooctanoate there was no difference between the two setups [corresponding estimate 1% (0, 3)]. Differences were negligible in the summer for all compounds.

**Conclusions:**

Transport of blood samples and processing delay, similar to conditions applied in some large, population-based studies, may affect measured perfluoroalkyl acid concentrations, mainly when outdoor temperatures are low. Attention to processing conditions is needed in studies of perfluoroalkyl acid exposure in humans.

## Introduction

Several studies have addressed potential health effects of human exposure to perfluoroalkyl and polyfluoroalkyl substances (PFASs), but not all studies have standardized the sampling and storage of biospecimens since PFASs are considered to have stable chemical structures [1]. Methods for exposure assessment differed in terms of type of biospecimen, processing and pre-analytic conditions (e.g. handling of samples before processing and freezing, storage temperatures, repeated freeze-thaw cycles), laboratory analyses, and the approach to levels below the limit of quantification (LOQ).

Although the extent of use of some PFASs [perfluorooctane sulfonate (PFOS) and perfluorooctanoate (PFOA)] is now limited by regulation in several countries [2–6], these compounds remain present in carpets, furniture, shoes, clothes, non-stick cookware, food packaging, and a variety of other consumer products, and similar chemicals are still used [7,8]. Of the large PFAS group, two perfluoroalkyl acids (PFAAs), PFOA and PFOS, are the most investigated compounds. PFOA and PFOS can be measured in biospecimens from most people and have serum half-lives of approximately 5 and 3.5 years, respectively [9]. Human exposure routes include ingestion, inhalation, and dermal absorption [10]. In pregnant women, PFOA and PFOS cross the placenta to the fetal circulation [11]. Serum and plasma are routine matrices in human studies, and the serum and plasma ratio approximates 1:1 for PFOS and PFOA [12].

Many studies on PFAAs have used material from large biobanks for which thousands of samples were collected and stored at very low temperatures (usually -80°C) for long periods of time before concentrations of PFAAs were measured. Some studies followed strict procedures allowing very short time between the collection of blood and processing and freezing [13,14] while others, e.g., the studies conducted in the Danish National Birth Cohort [11, 15–21] used samples that were transported for hours or up to two days as whole blood, at unknown temperatures, before processing and freezing. To our knowledge, the impact of such conditions prior to processing has not been evaluated in human whole blood samples. The aim of this study was to investigate whether measured plasma levels of PFAAs in paired samples from pregnant women differed between a setup that mimicked conditions in the DNBC and a setup of immediate processing and freezing, after both sets of plasma samples were stored for several years. We investigated whether any observed differences varied according to the season of blood sampling since we hypothesized that transportation temperatures could modify the final PFAA concentration.

## Methods

### Study design and participants

From March to August 2005, we recruited 88 pregnant women around gestational week 12 or 30 during routine antenatal visits at the Department of Obstetrics and Gynaecology, Aarhus University Hospital, Denmark. The study was designed to estimate the potential effect of delay and transportation under different outdoor temperatures (ranging from below 0˚C during the winter to above 20˚C during the summer). All women provided written informed consent prior to blood sampling.

### Ethics statement

Permission from the Health Research Ethics Committee, Central Denmark Region, was not required since the project was a validation study (journal number 205–2.0/6).

### Procedures

**Fig 1:**
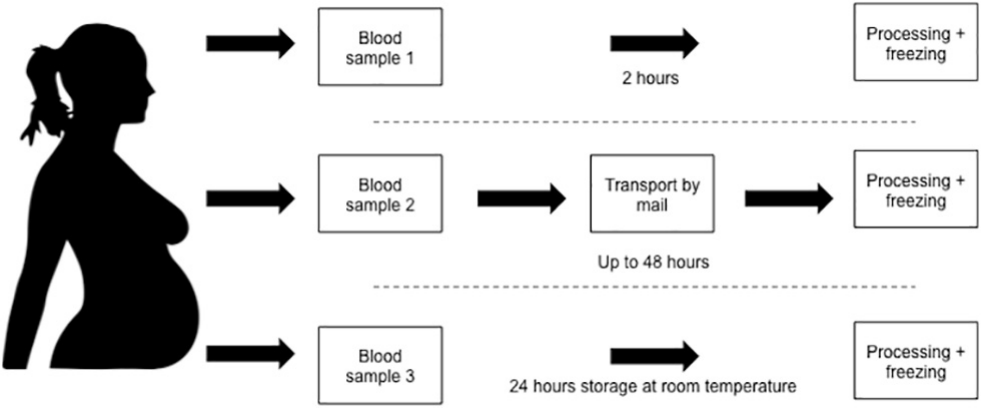
Illustration of the three designs applied to paired blood samples from pregnant women. Each woman donated two (n = 67) or three (n = 21) blood samples. For all women (n = 88), blood sample 1 and 2 were treated as displayed in the Fig. For the 21 women that provided a third blood sample this sample was treated as displayed for blood sample 3.

Each woman provided two (n = 67) or three (n = 21) samples of 9 mL blood that were drawn to EDTA tubes (Vacuette, Greiner Bio-One, Kremsmünster, Austria) at the same time and marked with encrypted identification numbers. The date and time were registered, and samples were treated according to three setups (Fig 1). In the immediate processing setup, 88 samples (36 during winter, 52 during summer) were processed, and plasma was frozen to -80˚C within two hours. The delay and transportation setup mimicked conditions for the DNBC sampling procedure (General practitioners took blood samples from pregnant women all over Denmark and mailed them to the cohort biobank in Copenhagen, where they were processed and plasma was frozen [15]). In our setup, we transported 88 samples at ambient temperature to one of four public mailboxes. Samples were returned to the laboratory by standard postal procedures after a maximum of 48 hours (most samples were returned within 24 hours). Samples were processed, and plasma was frozen upon arrival. A total of 21 women, who were all recruited during the summer, provided a third sample to which we applied delayed processing without transportation. These samples were left on a table in our laboratory at room temperature (approximately 20˚C) for 20–24 hours before they were processed and plasma was frozen in order to disentangle the effect of delayed processing in itself and delayed processing and transportation (including more various temperature changes and movements).

The processing method was identical for the three setups. Blood samples were centrifuged at 1730 g for 10 minutes at room temperature, and plasma was pipetted to polypropylene cryotubes (Sarstedt, Nümbrecht, Germany). In summary, 36 paired samples were available in the winter and 52 in the summer for comparison between immediate processing and processing delay and transportation. Twenty-one paired samples were available for the comparison between only delayed processing and delayed processing in combination with transport during the summer.

### PFAA analysis

Plasma samples from all three setups were stored at -80˚C until they were transported for approximately three hours to the Department of Environmental Science, Aarhus University, on dry ice in October 2013. A total of 12 PFAAs were measured [perfluorohexane sulfonate (PFHxS), perfluoroheptane sulfonate (PFHpS), PFOS, perfluorodecane sulfonate (PFDS), perfluorooctane sulfonamide (PFOSA), perfluoroheptanoic acid (PFHpA), PFOA, perfluorononanoic acid (PFNA), perfluorodecanoic acid (PFDA), perfluoroundecanoic acid (PFUnA), perfluorododecanoic acid (PFDoA), and perfluorotridecanoic acid (PFTrA)]. A laboratory technician blinded to sampling conditions performed all analyses. The extraction method was based on solid phase extraction [22]. Samples were spiked with ^13^C-labelled internal standards (10 ng/mL) before extraction [^13^C_2_-PFHxA, ^13^C_4_-PFOA, ^13^C_5_-PFNA, ^13^C_2_-PFDA, ^13^C_2_-PFUnA, ^13^C_2_-PFDoA, ^13^C_8_-PFOSA, ^18^O_2_-PFHxS, ^13^C_4_-PFOS, Wellington Laboratories (Guelph, ON, Canada)]. They were extracted and analyzed in batches with a procedural blank and two control samples. These consisted of test material analyzed in a ring test that had assigned values for PFOS and PFOA concentrations. The laboratory participated in the Arctic Monitoring and Assessment Programme (AMAP) Ring Test for Organic Pollutants in Human Serum organized by Institut National de Santé Publique du Québec to test method performance three times a year.

We used LC-MS-MS with electrospray ionization in negative mode for the analyses. The method detection limits (MDLs) were calculated as three times the standard deviation for blank values (n = 8). For compounds not having blank values, the MDL was calculated as three times the standard deviation of samples (n = 5) spiked to a concentration of 0.5 ng/mL (equal to five times the concentration of the lowest calibration standard).

The limit of quantification (LOQ) was calculated as 5 times the standard deviation for each compound. The precision of the method (intra-day and inter-day) was calculated at 1 ng/mL and 10 ng/mL spikes (see S1 Table). Five different samples were analyzed per day for four days by two different laboratory technicians. We determined the intra-day relative standard deviation % (RSD %) for each day as the standard deviation divided by the average level of the five samples multiplied by 100. The four intra-day RSDs were squared and added together, then divided by 4. Finally, the square root of this yielded the inter-day RSD %. In addition, we examined the precision of our method by analyzing 38 randomly selected duplicates from all three setups in order to compare the random variation in this setup with the potential systematic differences.

The measured bias for PFOS and PFOA was calculated from repeated analyses (n = 16) of human serum from one of the rounds of the AMAP Ring Test for Persistent Organic Pollutants in Human Serum (S1 Table). The values assigned for PFOS and PFOA by the organizer of the Ring test were used as certified values (other PFAAs were not included in the Ring Test before 2013).

### Statistical analysis

**Fig 2:**
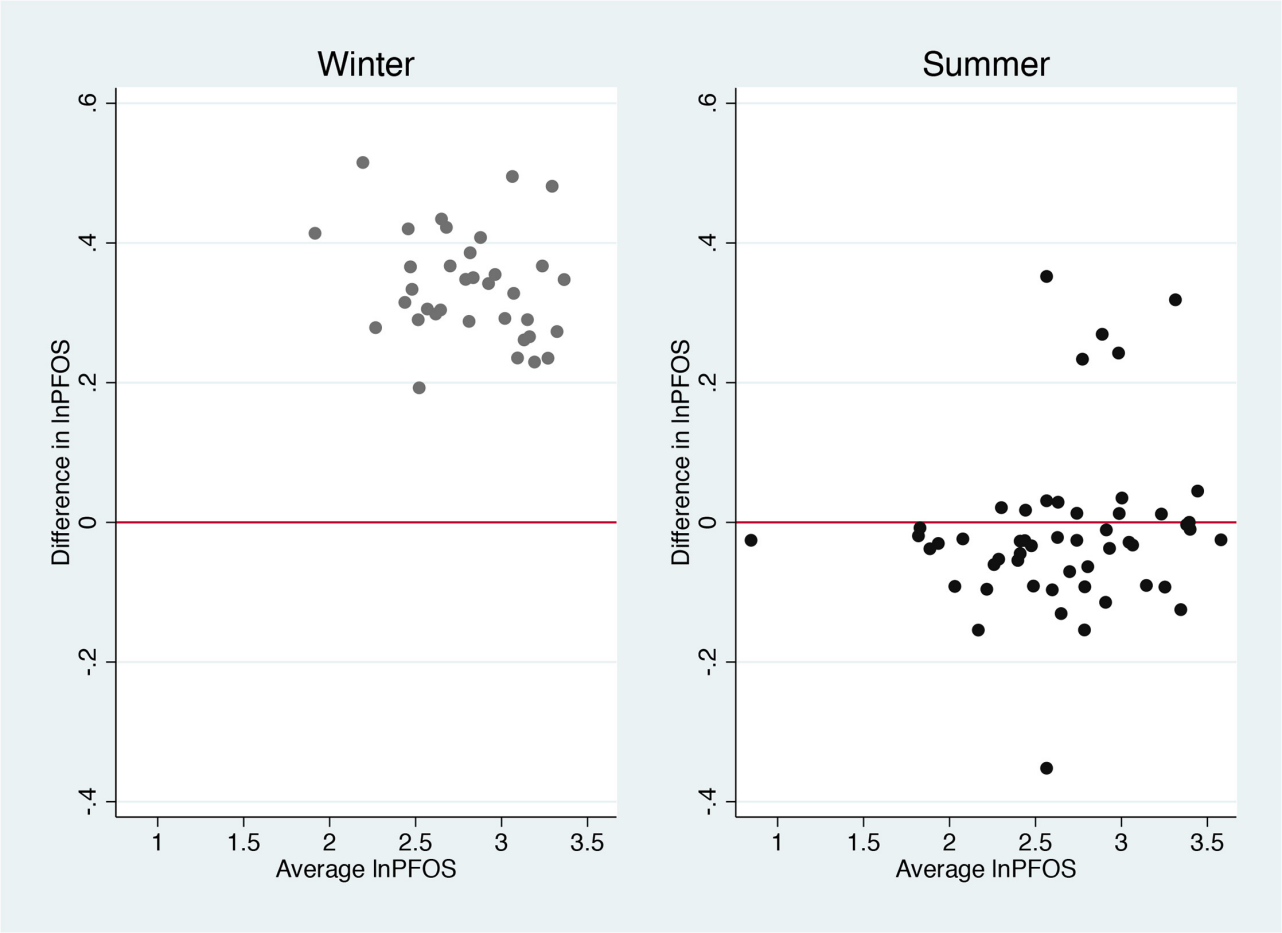
Bland Altman plot for perfluorooctane sulfonate after transformation by the natural logarithm, stratified by season. The first plot represents samples from the winter, while the second plot represents samples from the summer. The average lnPFOS is the average of the natural log-transformed concentrations of perfluorooctane sulfonate (PFOS) in the transported and delayed samples and the immediately processed samples. The difference is the difference between the two natural log-transformed concentrations.

We present results for PFAAs for which at least 50% of values were above the LOQ for the immediately processed samples. In the main analysis, all measured concentrations were included, also those below the LOQ. We used paired t-tests to investigate paired intra-individual differences for the immediately processed samples versus those with processing delay and transport. The analyses were done separately for winter and summer samples in order to assess differences according to different weather conditions for the transported samples. We assumed paired differences to be independent, and to test the other assumptions required for using paired t-test we performed histograms, Q-Q plots, Bland-Altman plots, and y = x plots [23]. For some of the investigated PFAAs, the differences increased with increasing average levels. Since this indicated a violation of one of the assumptions for using the paired t-test [23], and in order to identify relative instead of absolute differences, we transformed PFAA levels by the natural logarithm and repeated the plots (Fig 2), which then indicated no assumption violations. To ease the interpretation of the results we exponentiated the estimates from the paired t-tests, acquiring mean ratios. We present the mean ratios minus one to illustrate the relative paired differences in percent (for instance, a mean ratio of 1.10 is equal to a relative difference of 10%). Similarly, we compared the blood samples that were transported and delayed to those that were only delayed (n = 21).

We performed sensitivity analyses according to different approaches to values below the LOQ. First, we repeated the analyses using a common approach of substituting values below the LOQ with the LOQ divided by 2 [24, 25]. In another sensitivity analysis, we restricted to samples with concentrations above the LOQ. Multiple imputation of values below the LOQ was neither possible nor sensible in our setup. The 38 duplicates were analysed as described for the other paired comparisons. The statistical analyses were conducted in STATA statistical software version 12 (StataCorp, College Station, TX, USA).

## Results

For PFDoA, PFOSA, PFDS, and PFTrA > 50% of values were below the LOQ, and we do not present results for these compounds. The number of values below the LOQ for the included compounds is shown in Table 1. Median levels of the eight presented PFAAs ranged from 0.1 to 20 ng/mL in the immediately processed samples (Table 2).

**Table 1:** Perfluoroalkyl acids abbreviations, method detection limits, and limits of quantification.

PFAA	Full name	MDL (ng/mL)	LOQ (ng/mL)	N (%) below LOQ	
				Winter (n = 36)	Summer (n = 52)
PFUnA	Perfluoroundecanoic acid	0.05	0.15	0 (0)	8 (16)
PFDA	Perfluorodecanoic acid	0.03	0.09	0 (0)	0 (0)
PFHpS	Perfluoroheptane sulfonate	0.04	0.11	0 (0)	5 (10)
PFOS	Perfluorooctane sulfonate	0.09	0.28	0 (0)	0 (0)
PFNA	Perfluorononanoic acid	0.09	0.27	0 (0)	0 (0)
PFOA	Perfluorooctanoic acid	0.07	0.20	0 (0)	0 (0)
PFHxS	Perfluorohexane sulfonate	0.03	0.08	0 (0)	1 (2)
PFHpA	Perfluoroheptanoic acid	0.02	0.05	13 (36)	18 (35)

PFAA: Perfluoroalkyl acid

MDL: Method detection limit

LOQ: Limit of quantification.

n (%) below the LOQ applies to the immediately processed samples.

**Table 2:** Relative differences between PFAA concentrations in 88 paired blood samples that were immediately processed or transported with a processing delay, stratified by the season of sampling.

	Winter			Summer		
PFAA	Median_1_ (IQR)	Median_2_ (IQR)	Relative difference (95% CI)	Median_1_ (IQR)	Median_2_ (IQR)	Relative difference (95% CI)
	(ng/mL)	(ng/mL)	(%)	(ng/mL)	(ng/mL)	(%)
PFUnA	1.1 (0.8–1.3)	0.2 (0.2–0.3)	-77 (-78, -75)	0.3 (0.2–0.5)	0.3 (0.2–0.5)	-7 (-15, 3)
PFDA	0.5 (0.4–0.6)	0.3 (0.2–0.4)	-39 (-42, -3)	0.3 (0.2–0.4)	0.3 (0.2–0.4)	0 (-5, 5)
PFHpS	0.3 (0.2–0.4)	0.2 (0.1–0.3)	-41 (-47, -33)	0.2 (0.1–0.3)	0.2 (0.2–0.3)	7 (0, 14)
PFOS	20.0 (14.8–26.6)	14.3 (11.2–20.1)	-29 (-30, -27)	14.7 (10.8–20.6)	15.3 (11.1–19.8)	2 (-2, 5)
PFNA	0.8 (0.7–1.1)	0.7 (0.6–1.0)	-5 (-7, -3)	0.7 (0.6–1.0)	0.7 (0.6–1.0)	3 (0, 5)
PFOA	2.5 (1.9–3.8)	2.6 (2.0–3.9)	1 (0, 3)	2.5 (1.8–3.4)	2.5 (1.9–3.5)	3 (0, 6)
PFHxS	0.6 (0.5–0.7)	0.6 (0.5–0.8)	12 (3, 22)	0.6 (0.4–0.7)	0.6 (0.4–0.8)	11 (3, 19)
PFHpA	0.1 (0.0–1.1)	0.1 (0.0–0.2)	38 (12, 70)	0.1 (0.00–0.1)	0.1 (0.0–0.1)	17 (8, 27)

PFAA: Perfluoroalkyl acid

IQR: interquartile range

95% confidence interval (95% CI).

See Table 1 for specific PFAA abbreviations. Medians are not log-transformed.

Median_1_ refers to immediately processed samples. Median_2_ corresponds to samples that were delayed and transported and processing. 36 samples were sampled in the winter, and 52 in the summer.

For samples that were taken in the winter, relative PFAA differences between the delayed and transported samples and the immediately processed samples ranged between -77 and +38%. Five PFAAs (PFUnA, PFDA, PFHpS, PFOS, PFNA) were detected with lower concentrations in the delayed and transported samples [the relative difference (95% confidence interval) was -29 (-30, -27) for PFOS]. For PFOA, there was no difference between the two setups [relative difference (95% confidence interval) 1 (0, 3)], while for PFHxS and PFHpA, concentrations were higher in the delayed and transported samples. For samples taken during the summer, the relative differences between the immediately processed and the transported and delayed samples were negligible.

Concentrations were similar for the 21 paired samples from the summer when delayed processing was compared with delayed processing and transportation. Concentrations deviated by less than 10% for all compounds; for PFOS and PFOA relative differences (95% confidence interval) were -2 (-5, 1) and -1 (-3, 1), see Table 3.

**Table 3:** Relative differences between PFAA concentrations in 21 paired blood samples that were collected during the summer and transported after a processing delay or only had delayed processing without transport.

PFAA	Median_1_	Median_2_	Relative difference (95% CI)
	(ng/mL)	(ng/mL)	(%)
PFUnA	0.5	0.5	-3 (-8, 2)
PFDA	0.4	0.4	-3 (-7, 1)
PFHpS	0.3	0.3	-2 (-8, 6)
PFOS	17.1	16.1	-2 (-5, 1)
PFNA	0.8	0.8	0 (-3, 2)
PFOA	2.5	2.6	-1 (-3, 1)
PFHxS	0.7	0.6	-2 (-6, 3)
PFHpA	0.1	0.1	-9 (-16, -1)

PFAA: Perfluoroalkyl acid

for specific PFAA abbreviations see Table 1. Median_1_ refers to delayed and transported samples. Median_2_ corresponds to samples that were only delayed.

Sensitivity analyses restricted to values >LOQ or assigning these values equal to the LOQ divided by two provided results similar to the main analyses (S2 Table). Relative differences between duplicates were less than 3% for all PFAAs (S3 Table).

## Discussion

The present study was performed in order to investigate any relevant effect of transportation and processing delay on the measured concentration of substances in a set-up similar to many large-scale population studies. For most PFAAs, average concentrations in samples that were transported and had delayed processing during the winter differed from samples processed immediately. However, for samples that were transported and had delayed processing during the summer, concentrations were similar to those in samples that were processed immediately. We studied a sample of pregnant women, and our results may not be representative for other populations even though we do not expect pregnancy to modify the paired differences in the same woman.

Since we mimicked the DNBC setup as closely as possible, we did not control any of the factors than may be responsible for the observed differences in PFAA concentrations, such as indoor and outdoor temperatures or the exact amount of time in transport. Therefore we are not able to provide information on the mechanisms of the observed differences. Nonetheless, these findings are highly relevant to keep in mind when interpreting results from studies on PFAAs and health or disease outcomes in the DNBC or other cohorts using similar methods.

Some PFAAs were detected with higher and others with lower levels after transport and processing delay. Overall, lower concentrations were only seen in the samples from the winter, which were transported in cold weather (temperatures below 0˚C were present during that period). For the PFAAs with higher concentrations in transported samples, this observation was present both for samples in summer and winter.

Increased PFAA concentrations with transport and delayed processing may be due to conversion of precursor compounds. However, precursor compounds such as flurotelomer alcohols and sulfonamides are probably more likely to be metabolized to more stable acids like PFOS and PFOA in vivo than after blood sampling [26]. Evaporation is not likely to play an important role since the caps were closed during transportation. Since we used Teflon-free collection tubes, the risk of PFAA release from these was limited. Decreased concentrations may be caused by degradation of PFAAs, but this may not be very likely given the stability of the compounds. Adsorption to collection tubes can occur if polypropylene tubes are used [27]. However, we used polyethylene terephthalate collection tubes. In order to avoid adsorption, polyethylene or glass may be the preferable choice in terms of container material [27]. Binding of PFAAs to proteins, and therefore the measurable free fraction, might differ in unpredictable manners.

We used a validated laboratory method for all analyses, which minimized the risk of measurement error. Any measurement error would be non-differential since the laboratory technician, who performed the PFAA analyses, was blinded to conditions prior to analysis, and the sequence of samples was random. Our samples were stored for approximately 8 years at – 80° C, but paired samples were stored for equal amounts of time, and therefore potential measurement error due to storage would be non-differential. After initial freezing of the samples we did not thaw them until they were to be analysed.

The 24-hour PFAA stability at room temperature was high in rabbit serum and plasma [28]. Berger *et al*. [27] investigated the influence of storage for up to three months on PFAS stability in liquid chromatography-grade water stored in polypropylene containers at 4° C. In particular, long chained PFCAs such as PFUnA decreased in concentration (final recovery was less than 50%). Kato *et al*. [29] investigated the effects of storage temperature (room temperature, 5, -20 and -70˚C) and storage duration (up to eight months) on the stability of four PFAAs in 16 human serum samples. They found unchanged PFAA concentrations under the studied conditions, but they had no information on biospecimen collection, processing, and storage until arrival in their laboratory.

The results of our study should be replicated in other settings and laboratories. In future studies, it would be beneficial to investigate the influence of storage of fresh blood samples for various amounts of time at different temperatures (including temperatures below 0° C) on measured PFAA levels in different human matrices such as plasma, serum, whole blood, and blood cells, preferably using paired samples. The specific factors (such as the average temperature, temperature changes, and duration of transport or delay) and mechanisms behind any differences between the investigated setups should be clarified by use of a larger controlled design. We also encourage studies of dried blood spots on filter paper (this type of storage may potentially affect the stability of PFAAs) as well as cord blood, since results may be different from those presented due to the larger haematocrit and susceptibility to haemolysis in the newborn [30]. Similar studies concerning other chemicals would be of interest since chemicals with other properties, such as lipophilic persistent organic pollutants, may react differently to transportation and processing delay.

Our findings are relevant to studies that used samples subjected to delays and transport before processing, perhaps in particular in cold weather. According to our results, transportation in cold temperatures before processing may not affect PFOA much, but for PFOS, concentrations may decrease by approximately 30%. In studies where a delay in processing is unrelated to the exposure and outcome under study, measurement error may bias effect estimates towards null. However, if samples are collected throughout the year in a temperate climate, and the outcome under study is season-dependent, differential measurement error may arise and the estimate may be biased in an unpredictable direction. Adjusting for calendar time of blood drawing may therefore be important for studies where blood samples are not processed and frozen shortly after blood drawing.

## Conclusions

This study illustrates the need to consider the validity of laboratory data in studies where processing of biospecimens after blood drawing are less than perfect. Our results indicate that conditions in the pre-analytical phase may affect the measured concentration of PFAAs. However, these results should be replicated in a larger sample, and more studies are needed in order to determine which factors may be responsible for the observed differences.

## Supporting Information

S1 Dataset Concentrations of perfluoroalkyl acids in 88 plasma samples from pregnant Danish women, 2005. (XLSX)

S1 Table Precision for eight perfluoroalkyl acids and bias of the method. (DOCX)

S2 Table Sensitivity analyses of relative differences between perfluoroalkyl acid concentrations in immediately processed samples and samples with processing delay that were transported. (DOCX)

S3 Table Relative differences in perfluoroalkyl acid concentrations for 38 duplicate samples from all three setups. (DOCX)
