# Supplementary material for: Perfluoroalkyl Acid Concentrations in Blood Samples Subjected to Transportation and Processing Delay
Source: PLoS One. 2015 Sep 10;10(9):e0137768. doi: 10.1371/journal.pone.0137768 (PMC4565678; doi:10.1371/journal.pone.0137768)
Supplement: S1 Table — (DOCX) [file pone.0137768.s002.docx]

**S1 Table. Precision for eight perfluoroalkyl acids and bias of the method.**

| **Compound** | **Precision (RSD%)**  **1 ng/mL spike** | | **Precision (RSD%)**  **10 ng/mL spike** | | **Average concentration**  **± SD (ng/mL)** | **Assigned value (ng/mL)** | **RSD % of the assigned value** | **RSD% from the assigned value** |
| --- | --- | --- | --- | --- | --- | --- | --- | --- |
|  | **Inter-day** | **Intra-day** | **Inter-day** | **Intra-day** |  |  |  |  |
| PFHxS | 3.5 | 3.2; 5.1; 2.8; 2.1 | 1.2 | 1.3; 1.0; 1.1; 1.3 |  |  |  |  |
| PFHpS | 2.5 | 2.7; 0.7; 2.6; 3.3 | 2.7 | 1.8; 1.7; 4.6; 1.5 |  |  |  |  |
| PFOS | 7.7 | 14.4; 4.3; 2.8; 1.9 | 2.3 | 1.9; 2.0; 3.3; 1.4 | 36.6 (± 2.5) | 34.7 | 2.0 | 5.6 |
| PFHpA | 6.4 | 12.0; 3.0; 1.0; 2.7 | 1.9 | 1.7; 1.1; 2.6; 1.9 |  |  |  |  |
| PFOA | 2.6 | 2.1; 1.6; 1.7; 4.0 | 2.0 | 1.8; 2.2; 2.4; 1.5 | 8.1 (± 0.3) | 8.1 | 1.0 | 0.7 |
| PFNA | 3.5 | 6.2; 1.6; 1.7; 2.0 | 2.4 | 3.0; 1.3; 3.4; 1.0 |  |  |  |  |
| PFDA | 4.9 | 5.2; 4.1; 6.1; 3.7 | 3.1 | 2.4; 2.1; 4.2; 3.4 |  |  |  |  |
| PFUnA | 5.5 | 8.7; 1.7; 5.9; 3.0 | 5.1 | 4.8; 1.5; 8.5; 2.6 |  |  |  |  |

Abbreviations: Relative standard deviation (RSD), standard deviation (SD).

The bias of the method (RSD % of and from the assigned value) was calculated from repeated analyses of human serum (n=16), and is only available for PFOS and PFOA.
