# Supplementary material for: Perfluoroalkyl Acid Concentrations in Blood Samples Subjected to Transportation and Processing Delay
Source: PLoS One. 2015 Sep 10;10(9):e0137768. doi: 10.1371/journal.pone.0137768 (PMC4565678; doi:10.1371/journal.pone.0137768)
Supplement: S2 Table — (DOCX) [file pone.0137768.s003.docx]

**S2 Table. Sensitivity analyses of relative differences between perfluoroalkyl acid concentrations in immediately processed samples and samples with processing delay that were transported.**

|  |  |  | **Relative difference (95 % CI)**  **(%)** |  |  |
| --- | --- | --- | --- | --- | --- |
| **PFAA** | **Season** | **Pairs above the LOQ** | **All levels included** | **<LOQ encoded LOQ/2** | **Restricted to > LOQ** |
| PFUnA | Winter | 30 | -77 (-78, -75) | -78 (-80, -75) | -76 (-78, -74) |
|  | Summer | 44 | -7 (-15, 3) | -6 (-15, 4) | -9 (-19, 2) |
| PFDA | Winter | 35 | -39 (-42, -36) | -39 (-42, -36) | -39 (-42, -36) |
|  | Summer | 52 | 0 (-5, 5) | 0 (-5, 5) | 0 (-5, 5) |
| PFHpS | Winter | 30 | -41 (-47, -33) | -44 (-52, -35) | -35 (-41, -29) |
|  | Summer | 46 | 7 (0, 14) | 4 (-4, 12) | 6 (0, 14) |
| PFOS | Winter | 35 | -29 (-31, -27) | -29 (-31, 27) | -29 (-31, -27) |
|  | Summer | 52 | 2 (-2, 5) | 2 (-2, 5) | 2 (-2, 5) |
| PFNA | Winter | 35 | -5 (-7, -3) | -5 (-8, -3) | -5 (-7, -3) |
|  | Summer | 52 | 3 (0, 5) | 3 (0, 5) | 3 (0, 5) |
| PFOA | Winter | 35 | 1 (0, 3) | 1 (0, 3) | 1 (0, 3) |
|  | Summer | 52 | 3 (0, 6) | 3 (0, 6) | 3 (0, 6) |
| PFHxS | Winter | 35 | 12 (3, 22) | 12 (3, 22) | 12 (3, 22) |
|  | Summer | 51 | 11 (3, 19) | 10 (2, 18) | 10 (2, 19) |
| PFHpA | Winter | 23 | 38 (12, 70) | 34 (15, 57) | 31 (12, 53) |
|  | Summer | 34 | 17 (8, 27) | 14 (5, 24) | 12 (4, 21) |

Abbreviations: Lower limit of quantification (LOQ)

The column entitled “All levels included” is the main analysis for differences between the immediately processed samples and those with processing delay and transportation. The two subsequent columns present results from two sensitivity analyses; 1) encoding values below the LOQ with the LOQ divided by 2, and 2) restriction to values above the LOQ.
