# Supplementary material for: Perfluoroalkyl Acid Concentrations in Blood Samples Subjected to Transportation and Processing Delay
Source: PLoS One. 2015 Sep 10;10(9):e0137768. doi: 10.1371/journal.pone.0137768 (PMC4565678; doi:10.1371/journal.pone.0137768)
Supplement: S3 Table — (DOCX) [file pone.0137768.s004.docx]

**S3 Table. Relative differences in perfluoroalkyl acid concentrations for 38 duplicate samples from all three setups.**

| **Compound** | **Difference (95 % CI)**  **(%)** |
| --- | --- |
| PFUnA | 0 (-4, 3) |
| PFDA | 3 (0, 6) |
| PFHpS | 0 (-8, 8) |
| PFOS | 2 (0, 4) |
| PFNA | 0 (-2, 2) |
| PFOA | 2 (0, 3) |
| PFHxS | 2 (-3, 6) |
| PFHpA | 1 (-9, 9) |
